# Supplementary material for: Single molecule studies characterize the kinetic mechanism of tetrameric p53 binding to different native response elements
Source: PLoS One. 2023 Aug 15;18(8):e0286193. doi: 10.1371/journal.pone.0286193 (PMC10426914; doi:10.1371/journal.pone.0286193)
Supplement: S1 Fig — The blue lines are from the single exponential fits and the orange lines are from the double exponential fits. (PDF) [file pone.0286193.s001.pdf]

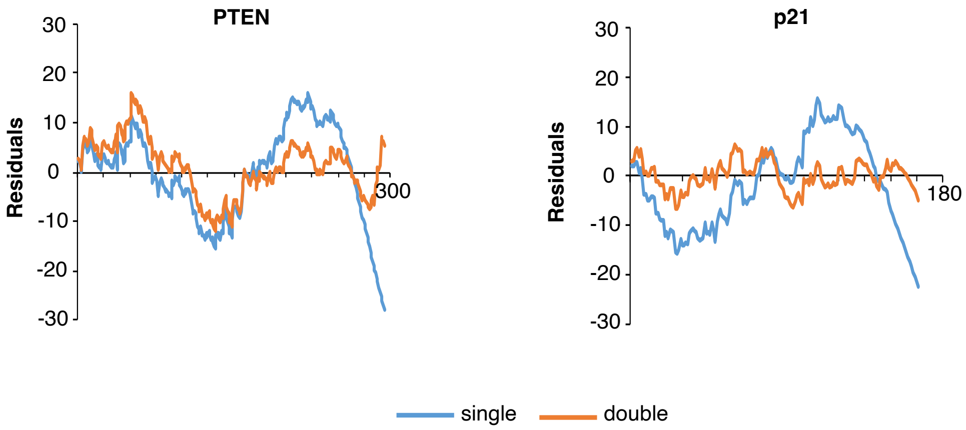

S1 Fig. Plots of residuals from the exponential fits of the unbound dwell times for PTEN and p21 REs. The blue lines are from the single exponential fits and the orange lines are from the double exponential fits.
