# Supplementary material for: Single molecule studies characterize the kinetic mechanism of tetrameric p53 binding to different native response elements
Source: PLoS One. 2023 Aug 15;18(8):e0286193. doi: 10.1371/journal.pone.0286193 (PMC10426914; doi:10.1371/journal.pone.0286193)
Supplement: S2 Fig — The blue lines are from the single exponential fits and the orange lines are from the double exponential fits. For all five REs the double exponential is a better fit for the data. (PDF) [file pone.0286193.s002.pdf]

Suwita, et al
Supporting Information
S2 Fig

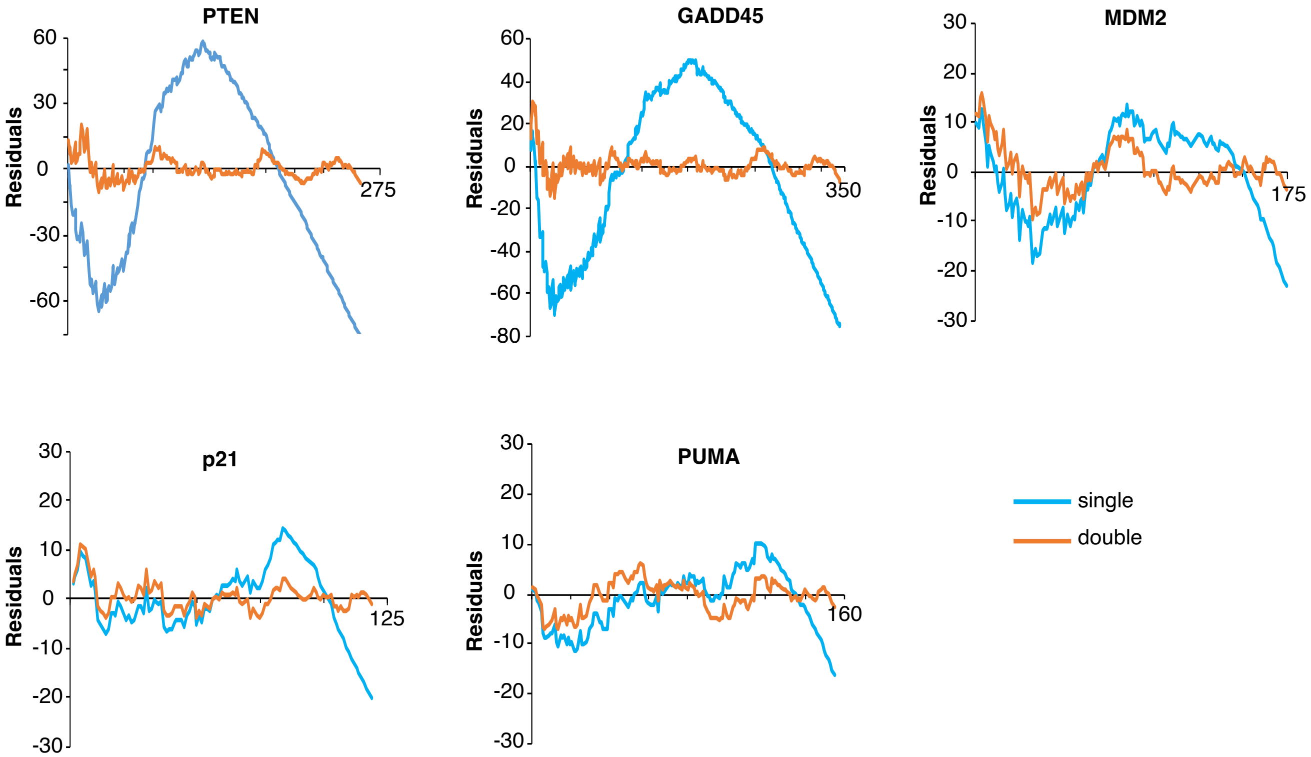

S2 Fig. Plots of residuals from the exponential fits of the unbound dwell times. The blue lines are from the single exponential fits and the orange lines are from the double exponential fits. For all five REs the double exponential is a better fit for the data.
